# Optimizing Fishery Survey Design in Guangdong’s Restricted Coastal Waters

**DOI:** 10.3390/ani15223283

**Published:** 2025-11-13

**Authors:** Kui Zhang, Li Su, Yancong Cai, Youwei Xu, Zuozhi Chen

**Affiliations:** 1 South China Sea Fisheries Research Institute, Chinese Academy of Fishery Sciences, Guangzhou 510300, China; zhangkui@scsfri.ac.cn (K.Z.); suli@scsfri.ac.cn (L.S.); caiyancong@scsfri.ac.cn (Y.C.); xuyouwei@scsfri.ac.cn (Y.X.); 2 Key Laboratory for Sustainable Utilization of Open-Sea Fishery, Ministry of Agriculture and Rural Affairs, Guangzhou 510300, China; 3 Guangdong Provincial Key Laboratory of Fishery Ecology and Environment, Guangzhou 510300, China

**Keywords:** fishery surveys, catch rates, species richness, sampling methods, dominant species

## Abstract

The coastal restricted fishing area of Guangdong contains key spawning and nursery habitats with high biodiversity but growing ecological pressure, yet the influence of survey design and sampling frequency on biodiversity detection and abundance estimates remains unclear. We conducted four seasonal bottom-trawl surveys in 2023–2024 at 186 stations and compared fixed-site sampling (FS), simple random sampling (SRS), stratified random sampling by depth (StRS), and systematic sampling (SS). We recorded 563 species (446 fishes, 101 crustaceans, 16 cephalopods), observed seasonal shifts in dominant taxa, and found catch rates varied seasonally and spatially, peaking in summer. Species detection rose with station number and sampling frequency. For species richness, SS produced the highest detection and the lowest error and bias but showed volatility; StRS and SRS were more stable. For abundance, StRS had the lowest error, whereas SRS had the smallest absolute bias. Across all four seasons, 88 stations achieved an 80% richness detection rate; among reduced-frequency designs, autumn-only, spring–autumn, and autumn–spring–summer minimized errors. These results guide cost–precision trade-offs: SS (with random starts and interval rotation) for richness-oriented aims, and depth-based StRS for abundance, supporting optimized long-term monitoring and management in the northern South China Sea.

## Simple Summary

Coastal waters in Guangdong are rich in marine life but face pressure from overfishing, pollution, and climate change. Managers need surveys that are both accurate and affordable. We carried out four seasonal bottom-trawl surveys at 186 sites inside the restricted fishing area and tested how different ways of choosing survey sites and survey timing affect results. We recorded 563 species and found clear seasonal and spatial changes, with the highest catches in summer. The more sites and the more seasons we sampled, the more species we found. A regularly spaced grid of sites detected the most species but could be unstable in some situations. Choosing sites at random within depth bands (shallow to deeper water) gave the most reliable estimates of how much fish was present. Sampling all four seasons required only 88 sites to find about four fifths of all species; if fewer surveys are possible, autumn alone, or spring and autumn together, performed best. These findings show how to design lean, effective surveys that stretch limited budgets while improving protection and management of key spawning and nursery habitats.

## 1. Introduction

The coastal waters of Guangdong, located on the continental shelf of the northern South China Sea (SCS), are characterized by high fisheries resource abundance and elevated biological diversity, influenced by the joint effects of Pearl River runoff, warm currents, and coastal upwelling [1,2]. In recent years, under multiple stressors—including overfishing, climate change, and marine pollution—the biomass of fishery resources in these waters has declined considerably, accompanied by increasing ecosystem instability [3,4]. Notably, since the 1980s, intensified fishing pressure has led to the continual depletion of important commercial species, including largehead hairtail (*Trichiurus lepturus*) [5,6].

**Figure 1:**
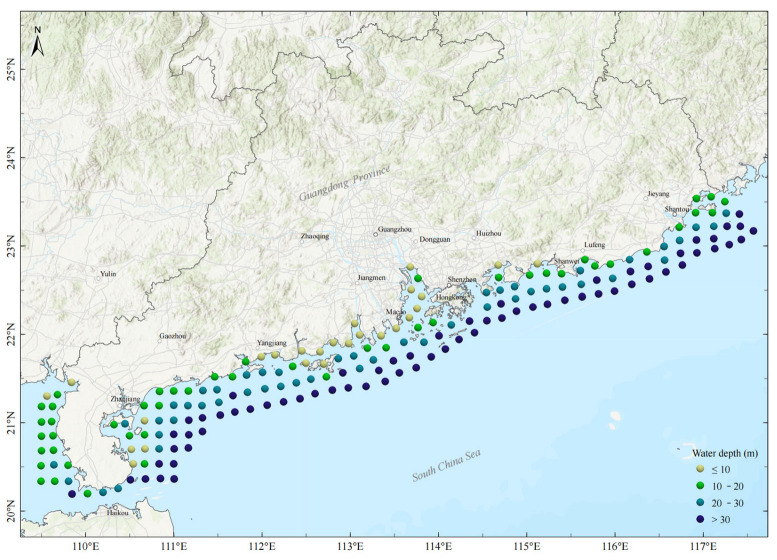
Survey sites with bathymetric information. The area covered by the survey sites is the restricted fishing area in Guangdong’s coastal waters.

Spawning and nursery grounds serve as critical habitats that support natural recruitment. By ensuring a consistent supply of new individuals into the population, they directly mitigate overexploitation and alleviate ecological stress on fisheries [7,8]. Therefore, since 1990, the Chinese government has designated restricted fishing area in coastal shallow-water areas that function as key spawning and nursery habitats for numerous economically important fish species [9]. Motorized trawling is strictly prohibited within these boundaries. The restricted fishing area in Guangdong (Figure 1) encompasses several vital fish habitats—including Daya Bay, the Pearl River Estuary, and Leizhou Bay—which are recognized for high biodiversity and significant fishery resources. Nevertheless, these ecosystems and associated fish communities have undergone substantial changes under persistent stressors such as climate change, pollution, and engineering construction. These changes are manifested through biodiversity loss, simplification of community structure (including a decline in mean trophic level), and a regime shift from communities dominated by high-value, demersal species to those dominated by smaller, pelagic, and more resilient species [3,10,11]. Fisheries surveys conducted in the restricted fishing area have predominantly focused on a limited number of these key ecosystems and are lacking in spatially comprehensive investigations. Consequently, there is limited understanding of the current species composition, dominant species, and abundance distributions within fishery resources, thereby impeding effective conservation and scientific management.

Fisheries monitoring and sustainability are critically dependent on accurate assessments of resource diversity and biomass, which in turn rely on the quality and robustness of data derived from scientifically grounded sampling programs [12,13]. The methodology underlying sampling design in fisheries surveys has evolved from traditional empirical strategies to modern, optimized approaches. Currently, mainstream sampling design methods include fixed-site sampling (FS), simple random sampling (SRS), stratified random sampling (StRS), systematic sampling (SS), and cluster sampling (CS), each with distinct strengths and limitations demonstrated by numerous studies [14,15,16,17]. Research indicates that StRS can enhance sampling accuracy and stability through scientific stratification and rational station allocation [18,19]. Fishery monitoring and sustainability are critically dependent on accurate assessments of resource diversity and biomass, which in turn rely on the quality and robustness of data derived from scientifically grounded sampling programs [20]. Therefore, the selection of an appropriate survey method requires comprehensive consideration of both the spatial heterogeneity of fishery resources and the underlying research objectives. Additionally, sampling effectiveness is inherently linked to sample size: larger sample sizes generally yield more accurate and representative survey data [21]. In practice, however, consideration must also be given to constraints of cost, time, and human resources, necessitating the identification of an optimal trade-off between sample size, efficiency, and data precision.

During 2023–2024, we conducted the first comprehensive fishery resource survey in the restricted marine fishing area of Guangdong, setting up 186 survey sites (Figure 1) and using bottom trawling to evaluate the basic status of fishery resources in this area, including species composition and resource abundance. However, due to insufficient funding and manpower, it will not be possible for subsequent surveys to follow the same frequency and number of sites, and they will definitely be scaled down. Therefore, it is urgent to carry out research on optimizing sampling for fishery surveys to ensure the scientific validity and accuracy of the survey results. Based on four-season bottom trawl data collected from 2023 to 2024, this study systematically evaluates—for the first time—how sampling design (FS, SRS, StRS, and SS) and sample size influence species detection and abundance estimation in this region, with the goal of providing a scientific foundation for fishery conservation and evidence-based management.

## 2. Materials and Methods

### 2.1. Survey Data

The study area (Figure 1) encompasses the coastal waters of Guangdong Province, spanning water depths from 0 to 40 m. A total of 186 predetermined survey stations were established within the restricted fishing zone, comprising 25 stations at depths ≤10 m, 47 at depths >10 m and ≤20 m, 48 at depths >20 m and ≤30 m, and 66 at depths >30 m.

Field surveys were conducted using bottom trawling, with four seasonal voyages undertaken in autumn (November 2023), winter (December 2023 to January 2024), spring (March to April 2024), and summer (August to September 2024). All four surveys utilized the same bottom trawling vessel equipped with a 200 mm main mesh and a 40 mm cod-end mesh, ensuring sampling consistency across voyages. At most stations, the trawl net was towed at an average speed of 3.0 knots for 30 min. However, at approximately 5% of the stations—specifically those in extremely shallow waters or areas with intense maritime traffic—the towing duration was reduced to 20–25 min as a preventive measure against collisions with other vessels or fixed fishing gear.

At each station, all captured fish specimens were taxonomically identified to species [22,23]. For each species, both the total number of individuals and the catch weight were recorded. Species richness was represented by the number of species, while the relative abundance at each station was calculated as the total catch weight divided by trawling time (kg/h). The catch density (*D*, kg/km^2^) was derived as *D* = *C*/(*v* · *t* · *L* · *X*), with *C* being catch biomass (kg), *v* the trawl speed (km/h), *t* the tow duration (h), *L* the headrope length (km), and *X* the effective fishing width factor, for which a standard value of 0.66 was used.

### 2.2. Dominant Fish Species

The ecological dominance of species was calculated using the index of relative importance (IRI) [24]:

IRI = (N + M) × F

where *N* and *M* are the percentages of abundance of a specific species relative to the total abundance of all species, by number and by weight, respectively; and *F* is the frequency of occurrence, defined as the proportion of sampling stations at which the species was present (i.e., the number of stations where the species occurred divided by the total of 186 stations). The higher the IRI value, the greater is the relative importance of the species. Species were considered dominant when IRI >  500 [25].

### 2.3. Sampling Design

#### 2.3.1. Fixed-Site Sampling

The Fixed-Site Sampling (FS) scenario was simulated by first randomly establishing a fixed set of *n* stations (for each *n* from 8 to 184) from the total 186. This predetermined set was then used consistently across all simulation replicates for that sample size, emulating a long-term survey that revisits the same randomly located stations.

#### 2.3.2. Simple Random Sampling

A random selection with replacement [26] was performed among the 186 survey stations, sequentially drawing samples of 8, 16, 24, …, 168, 176, and 184 stations (in increments of 8). For each sample size, the sampling process was repeated 1000 times.

#### 2.3.3. Stratified Random Sampling

The survey stations were divided into four layers based on water depth (A: <10 m; B: 10–20 m; C: 20–30 m; D: >30 m). For each sample size, the number of stations selected from each layer was allocated proportionally to the number of stations within that layer [26]. Within each layer, stations were selected using random sampling. The total sample sizes were consistent with those used in FS and SRS. For each sample size, the sampling process was repeated 1000 times.

#### 2.3.4. Systematic Sampling

The 186 stations were first arranged according to station ID, with the first starting station selected via simple random sampling [27]. The remaining stations were then selected in sequence at fixed intervals. The total sample sizes were consistent with those used in FS and SRS. For each sample size, the sampling process was repeated 1000 times.

#### 2.3.5. Sampling Frequency

Fifteen seasonal combination patterns were set up, including once per year (summer, autumn, winter, spring), twice per year (summer–autumn, summer–winter, summer–spring, autumn–winter, autumn–spring, winter–spring), three times per year (summer–autumn–winter, summer–autumn–spring, summer–winter–spring, autumn–winter–spring), and four times per year (summer–autumn–winter–spring), to evaluate the effect of sampling frequency on species richness metrics.

Relative abundance was evaluated only for the effect of different sampling methods on the results across the four seasons.

### 2.4. Evaluation of Sampling Design Effects

#### 2.4.1. Species Richness Detectability

Species richness detectability for fishery species is defined as the proportion of species obtained in a sampling survey to the “true” number of species in the surveyed sea area. The calculation formula of detection rates is as follows [28]:

$$P\;=\;S/S_{\mathrm{true}}\;\times \;100\%$$

where *P* is the species richness detectability and *S* is the number of species obtained in a resampling survey. *S*_true_ is the “true” number of species in the surveyed sea area, and is the total species number obtained in all four surveys in this study.

#### 2.4.2. Relative Estimation Error

The relative estimation error (REE) is used to evaluate the precision and accuracy of the estimated value (detection rates of species richness, and relative abundance). The calculation formula is as follows [29]:

$$\mathrm{REE}\;=\;\frac{\sqrt{\sum_{i\;=\;1}^{R}{\left({O_{i}}^{\mathrm{estimated}}-O^{\mathrm{true}}\right)}^{2}/R}}{O^{\mathrm{true}}}\;\times \;100\%$$

where *O*^ture^ is the true value of number of species or relative abundance in the surveyed sea area, *O* iestimated is the number of fish species obtained in the ith resampling, and *R* is the number of resampling times (1000 in this study).

#### 2.4.3. Relative Bias

Relative bias (RB) can be used to evaluate the accuracy of survey estimates and their deviation from the true value. The calculation formula is as follows [29,30]:

$$\mathrm{RB}\;=\;\frac{\sum_{i\;=\;1}^{R}{O_{i}}^{\mathrm{estimated}}/R-O^{\mathrm{true}}}{O^{\mathrm{true}}}\;\times \;100\%$$

Smaller absolute values of REE and RB indicate more accurate assessment results.

### 2.5. Statistical Analysis

The significance analysis of the data in the text first conducts analysis of variance (ANOVA) and homogeneity of variance test (Levene’s Test). If the ANOVA is significant, post hoc multiple comparisons (Tukey HSD) are carried out, with the significance criterion being *p* < 0.05.

The R language code used in this study is detailed in the Supplementary Materials.

## 3. Results

### 3.1. Species Composition and Dominant Species

Across the four seasonal surveys conducted from 2023 to 2024, a total of 563 fishery species were captured, comprising 446 fish species (79.2%), 101 crustacean species (17.9%), and 16 cephalopod species (2.8%). This total number of observed species (N = 563) was used for the subsequent analysis of species richness detectability.

Dominant species exhibited pronounced seasonal shifts (Table 1). Notably, the deep pugnose ponyfish (*Deveximentum ruconius*) was a common dominant species across spring, summer, and autumn. In addition, three other ponyfish species (Leiognathidae)—the shortnose ponyfish (*Leiognathus brevirostris*), orangefin ponyfish (*Photopectoralis bindus*), and berber ponyfish (*Equulites berbis*)—also appeared on the list of dominant species in different seasons.

Table 1 The dominant species and their IRI (the values in the brackets) in the study area for each of four seasons during 2023–2024.

### 3.2. The Temporal and Spatial Variations of Fishery Resources

**Figure 2:**
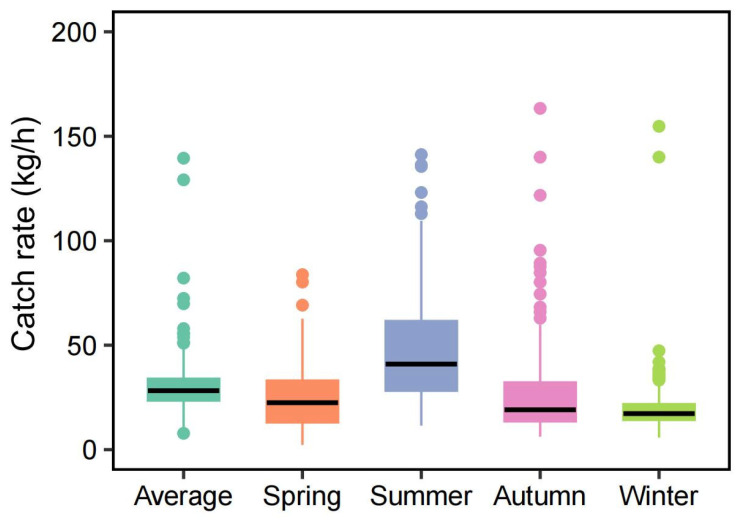
The box plots of catch rates of fisheries resources for the four seasons in the study area.

The catch rates of fishery resources exhibited clear seasonal variation (Figure 2). The seasonal mean catch rates (±SD) were as follows: spring, 24.44 ± 13.94 kg/h; summer, 48.74 ± 35.05 kg/h; autumn, 29.80 ± 43.49 kg/h; and winter, 20.73 ± 15.15 kg/h. Specifically, the catch rate in summer was significantly higher than in the other seasons (*p* < 0.001), and in spring it was significantly higher than in winter (*p* < 0.01). However, no significant differences were observed between spring and autumn (*p* > 0.05) or between autumn and winter (*p* > 0.05).

**Figure 3:**
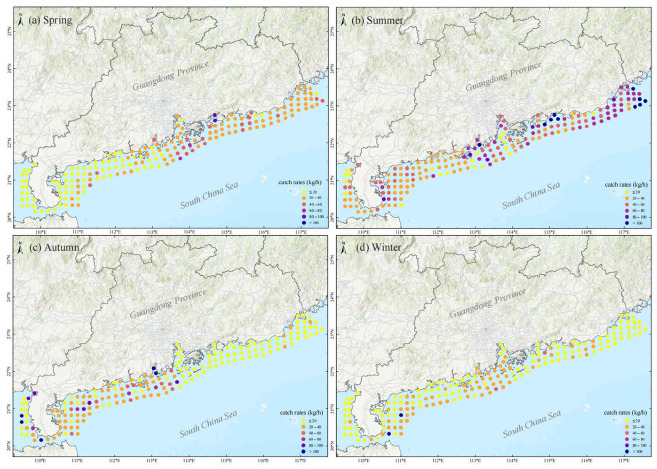
Catch rates of fishery resources at each station across the four seasons.

Catch rates also exhibited marked spatial variations (Figure 3). In spring, the high-value zones were located at the outermost station near the Pearl River Estuary (54.94 ± 10.57 kg/h) and Daya Bay (81.99 ± 2.51 kg/h), and catch rates in the eastern Guangdong waters (34.91 ± 12.00 kg/h) were significantly higher than in the western Guangdong waters (16.93 ± 9.83 kg/h); in summer, the high-value zones were located in Honghai Bay (123.42 ± 86.33 kg/h), around Nan’ao Island (110.49 ± 20.67 kg/h), the Wanshan Archipelago (66.93 ±13.84 kg/h), and adjacent waters; in autumn, the high-value zones were more dispersed, and catch rates in the western Guangdong waters (39.51 ± 50.81 kg/h) were significantly higher than in the eastern Guangdong waters (13.72 ± 4.12 kg/h); in winter, the high-value zones were located in the western waters of the Pearl River Estuary (30.48 ± 7.05 kg/h) and the eastern waters off the Leizhou Peninsula (40.10 ± 50.82 kg/h).

### 3.3. Species Richness

#### 3.3.1. Detection Rates

**Figure 4:**
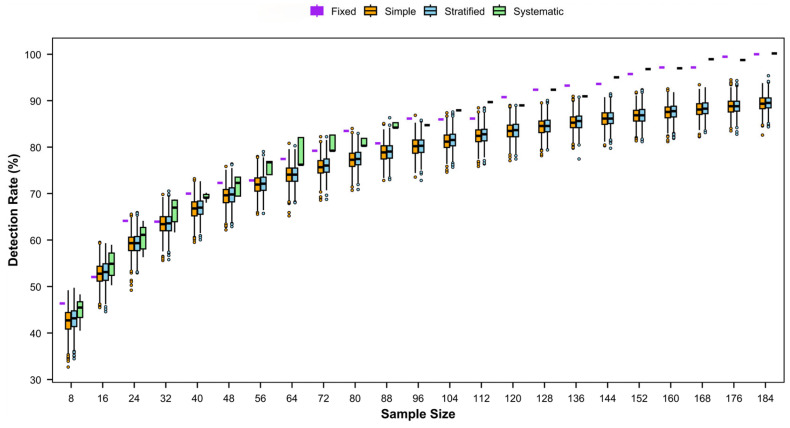
Detection rates of fishery species with different sampling designs by sampling frequency of four times (spring–summer–autumn–winter) per year.

**Figure A1:**
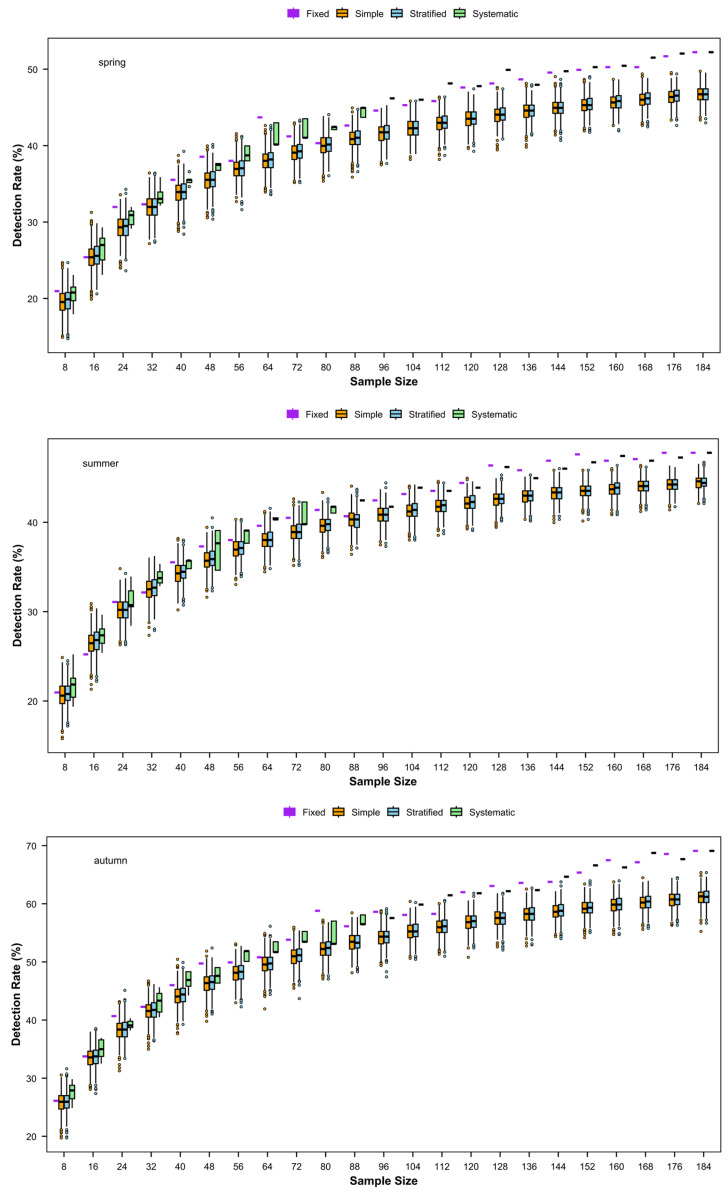

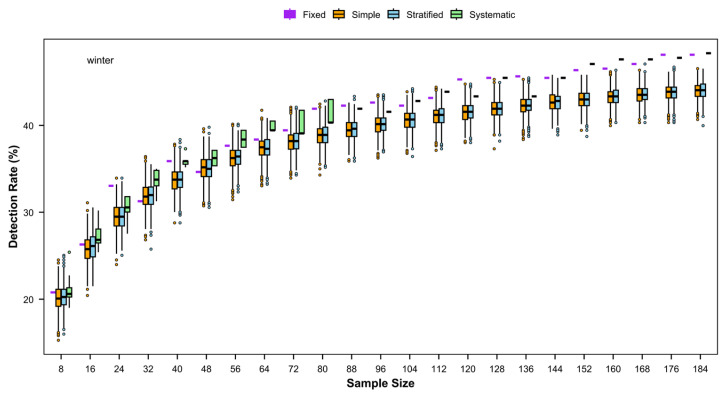
Detection rates of species richness of fishery species with different sampling designs by sampling frequency of once per year.

**Figure A2:**
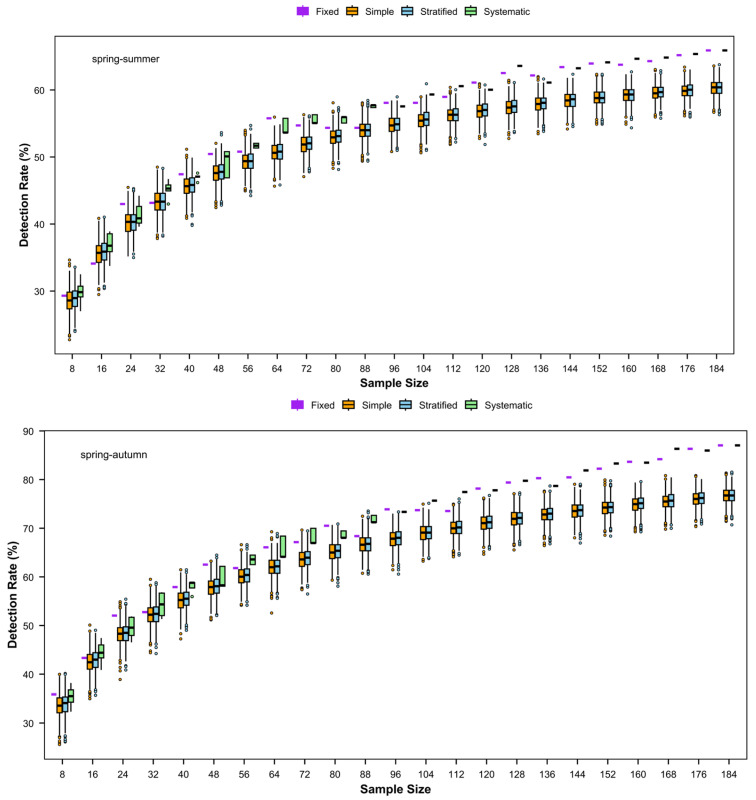

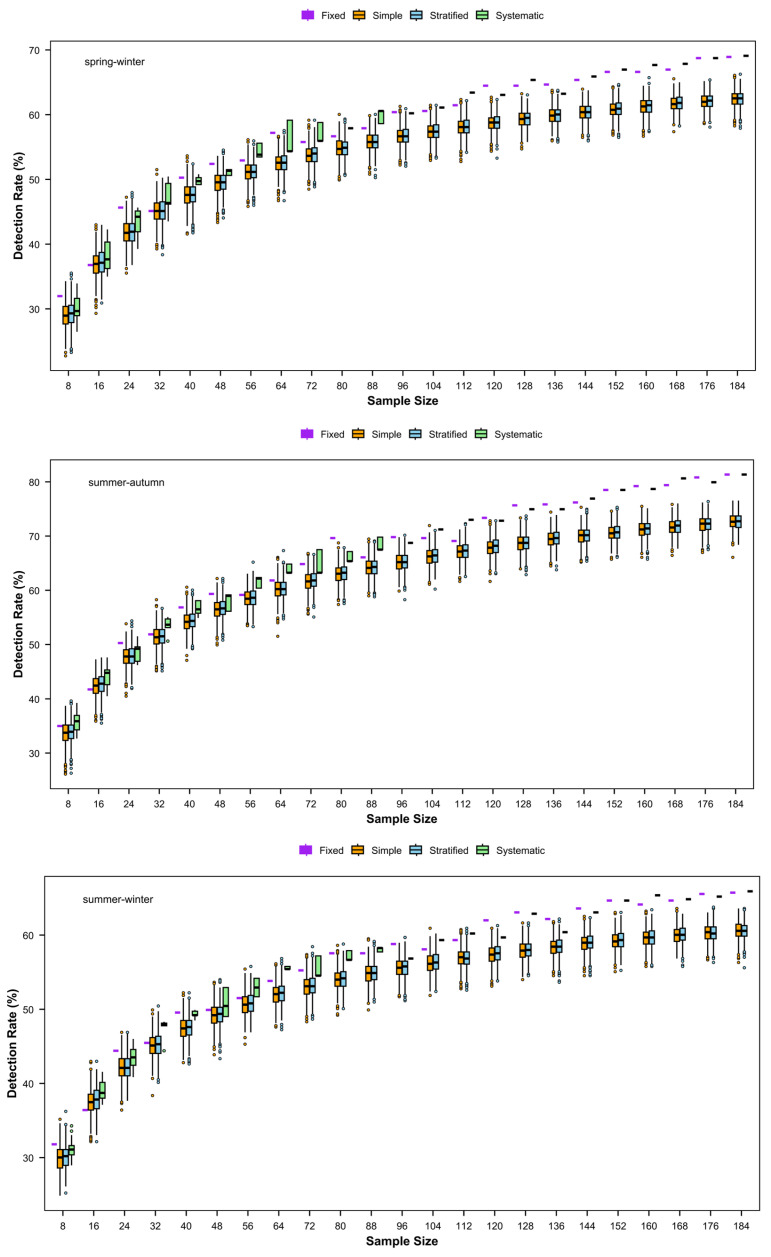

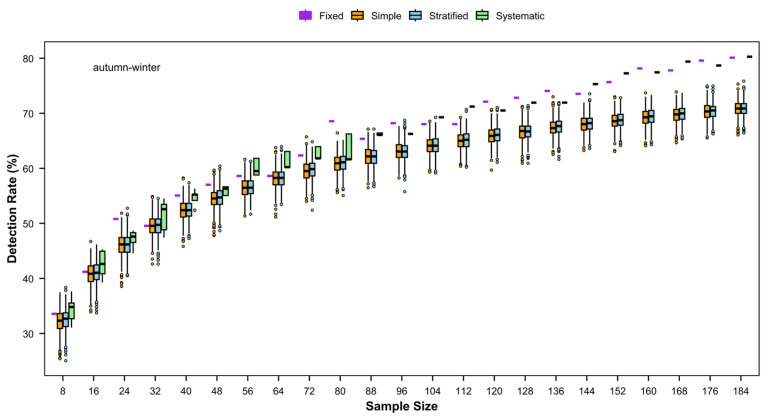
Detection rates of species richness of fishery species with different sampling designs by sampling frequency of twice per year.

**Figure A3:**
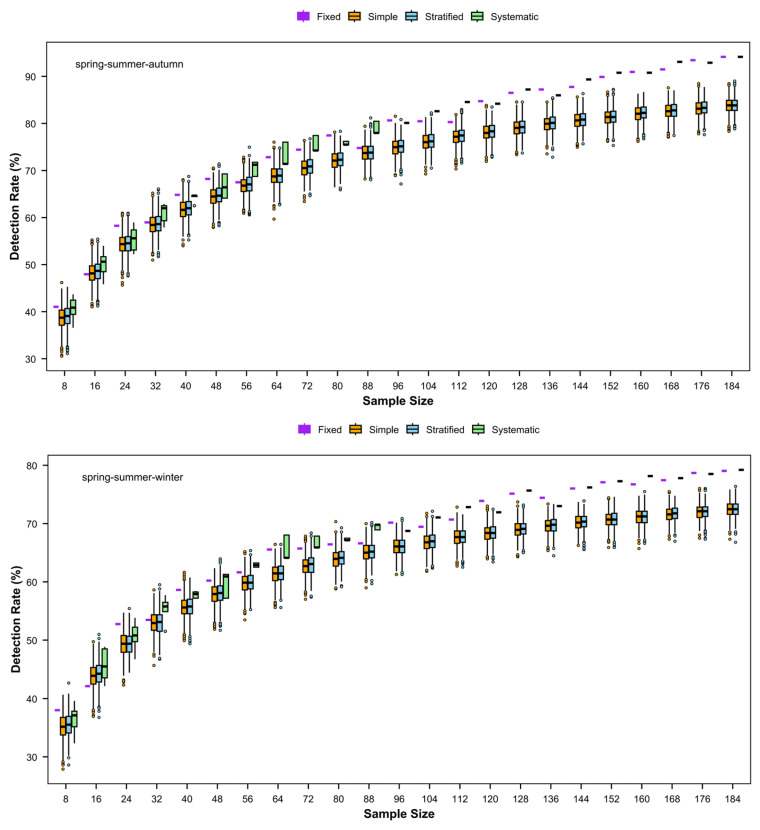

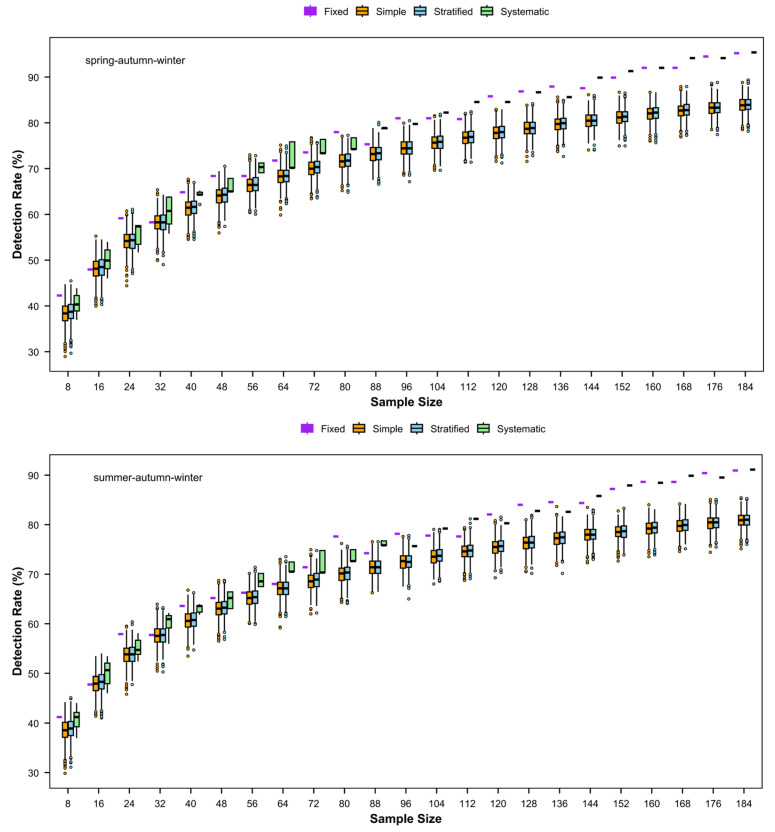
Detection rates of species richness of fishery species with different sampling designs by sampling frequency of three times per year.

Across all sampling designs, the detection rate of species richness increased progressively with the number of stations (Figure 4, Appendix A Figure A1, Figure A2 and Figure A3). When the number of stations was small, the detection rate rose rapidly; as the number of stations increased, the rate of increase gradually stabilized.

Detection rates increased consistently with higher sampling frequency (Figure 4, Appendix A Figure A1, Figure A2 and Figure A3). When survey stations were held constant, more frequent sampling yielded higher detection rates. For a given sampling frequency, sampling efficiency differed among seasonal combinations: with one survey per year (Appendix A Figure A1), autumn performed best (detection rates for all four sampling designs were highest among four seasons); with two surveys per year (Appendix A Figure A2), the spring–autumn combination was optimal (requiring only 112 stations for all four designs to exceed 70%); with three surveys per year (Appendix A Figure A3), the autumn–spring–summer combination performed best (requiring only 136 stations for all four designs to exceed 80%). When sampling across all four seasons, the systematic sampling method only 88 stations were required to achieve an 80% detection rate (Figure 4).

Detection rates differed among sampling methods. Systematic sampling achieved the highest detection rates but exhibited large fluctuations, indicating unstable performance. Fixed-site sampling was slightly lower than systematic sampling and also shows large fluctuations. Simple random sampling and stratified random sampling showed more stable detection performance, with stratified random sampling outperforming simple random sampling.

#### 3.3.2. REE of the Detection Rates

**Figure 5:**
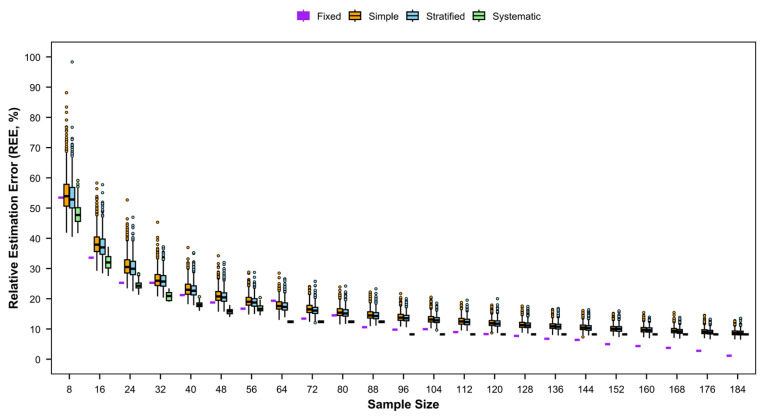
Relative estimation error (REE) of detection rates of fishery species with different sampling designs by sampling frequency of four times (spring–summer–autumn–winter) per year.

**Figure A4:**
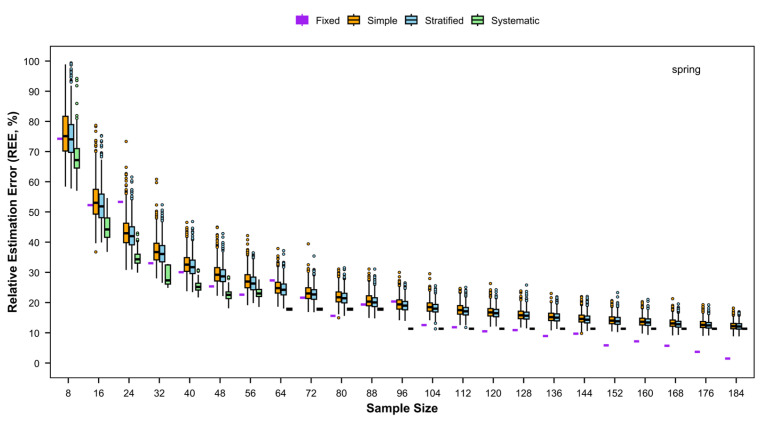

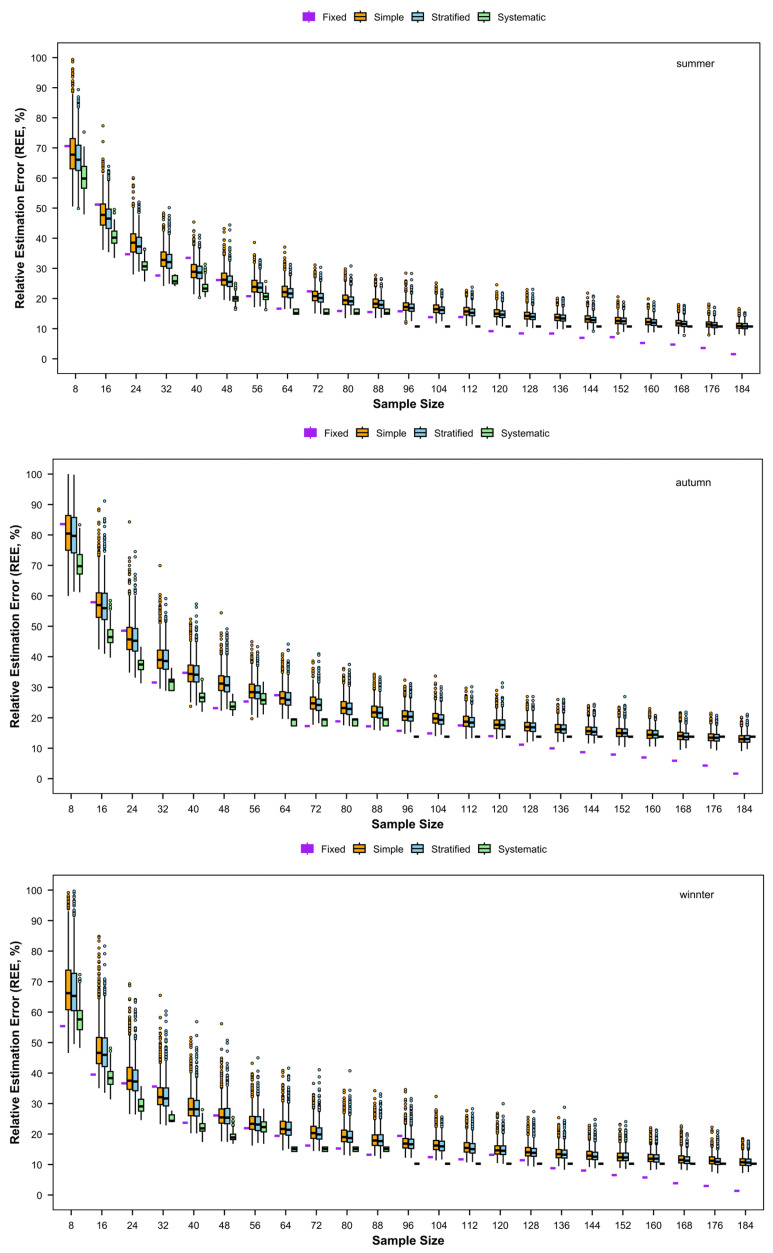
Relative estimation error (REE) of detection rates of fishery species with different sampling designs by sampling frequency of once per year.

**Figure A5:**
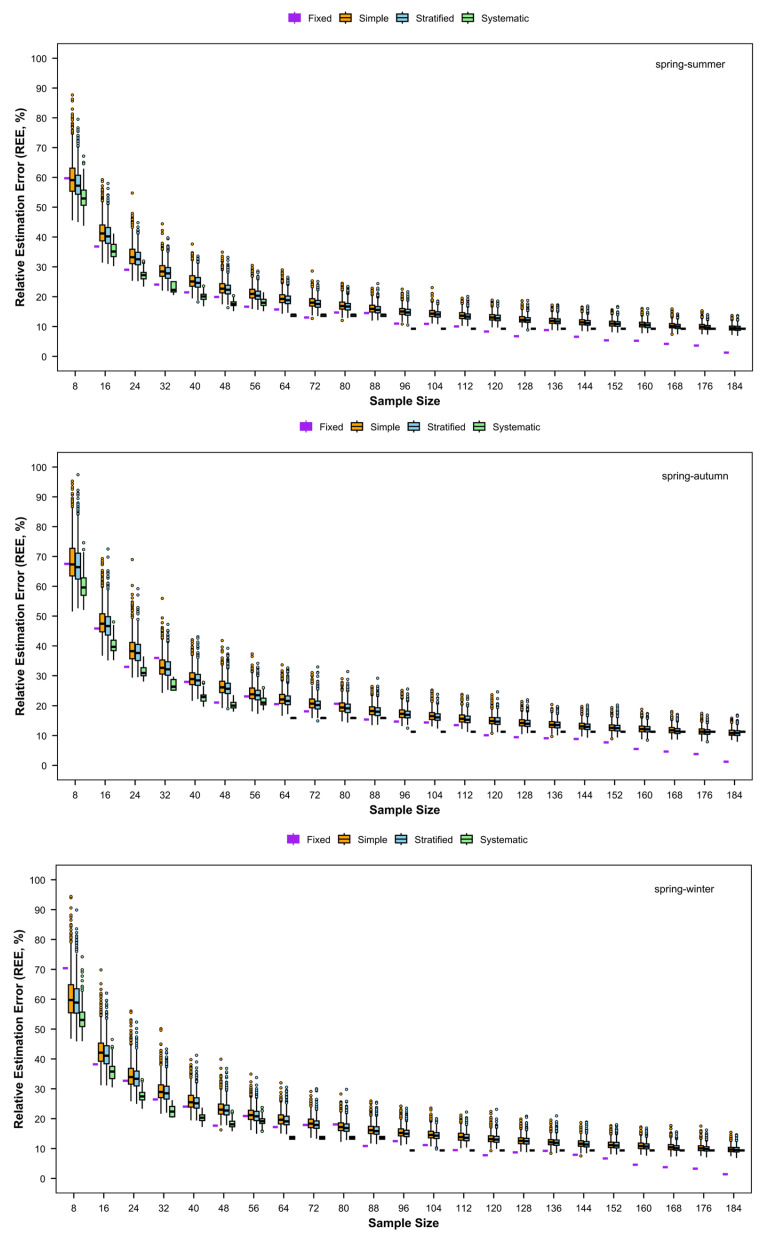

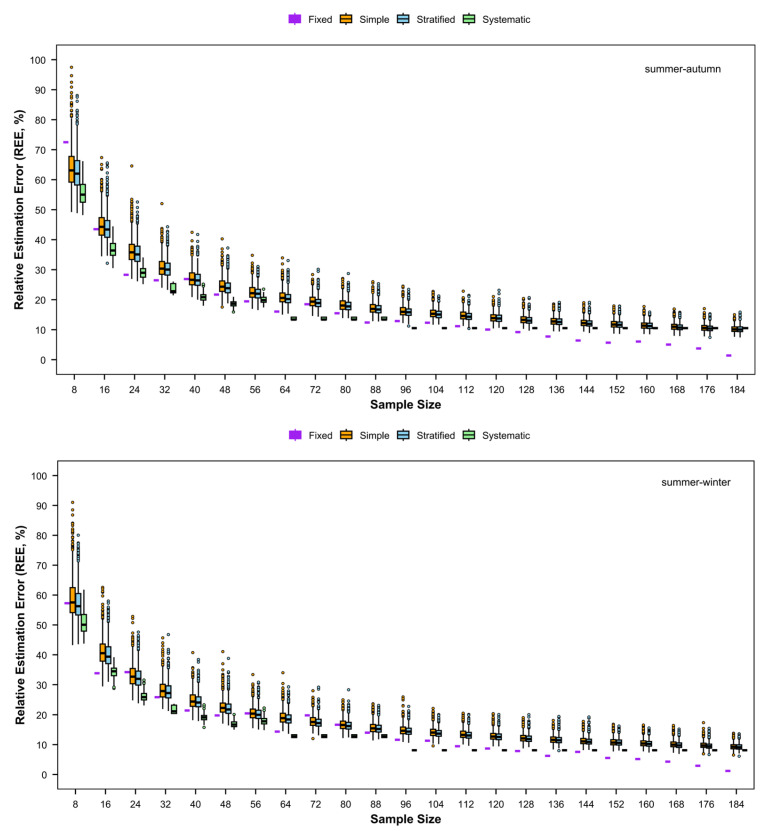
Relative estimation error (REE) of detection rates of fishery species with different sampling designs by sampling frequency of twice per year.

**Figure A6:**
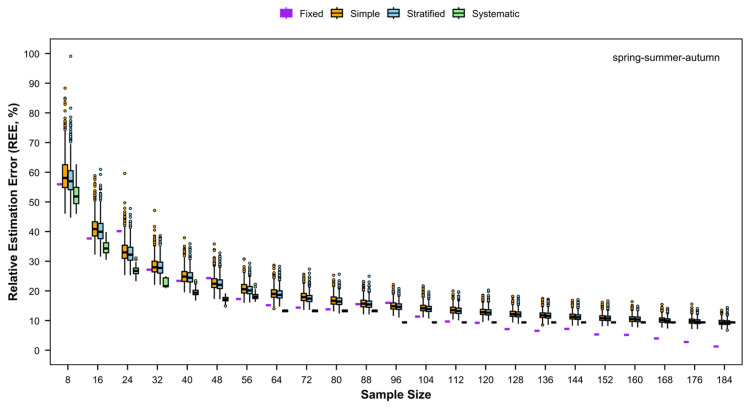

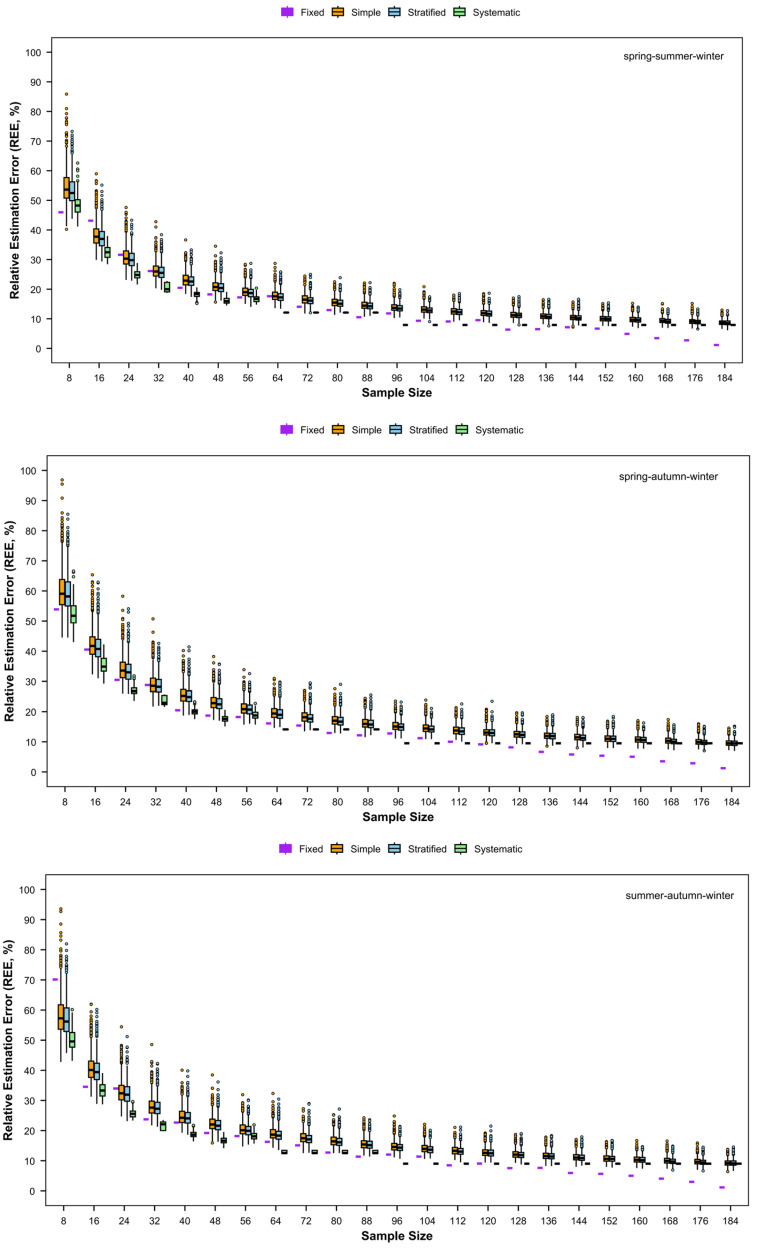
Relative estimation error (REE) of detection rates of fishery species with different sampling designs by sampling frequency of three times per year.

For all sampling designs, the REE of species richness detection rates decreased overall as the number of stations increased (Figure 5, Appendix A Figure A4, Figure A5 and Figure A6). The decline was faster at smaller sample sizes and then leveled off. Among methods, systematic sampling had the smallest REE, followed by fixed-site sampling, while simple random sampling had the largest REE. As the number of stations increased, the REE difference between stratified random sampling and simple random sampling became increasingly smaller.

REE varied with sampling frequency. Higher sampling frequency resulted in smaller REE for a given number of stations. When sampling once, twice, and three times per year, the autumn-only (Appendix A Figure A4), spring–autumn (Appendix A Figure A5), and autumn–spring–summer (Appendix A Figure A6) combinations, respectively, produced the smallest REE.

#### 3.3.3. RB of the Detection Rates

**Figure 6:**
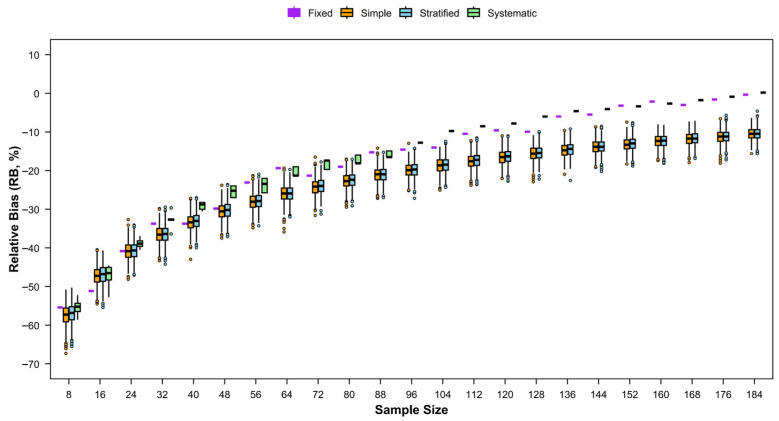
Relative bias (RB) of detection rates of fishery species with different sampling designs by sampling frequency of four times (spring–summer–autumn–winter) per year.

**Figure A7:**
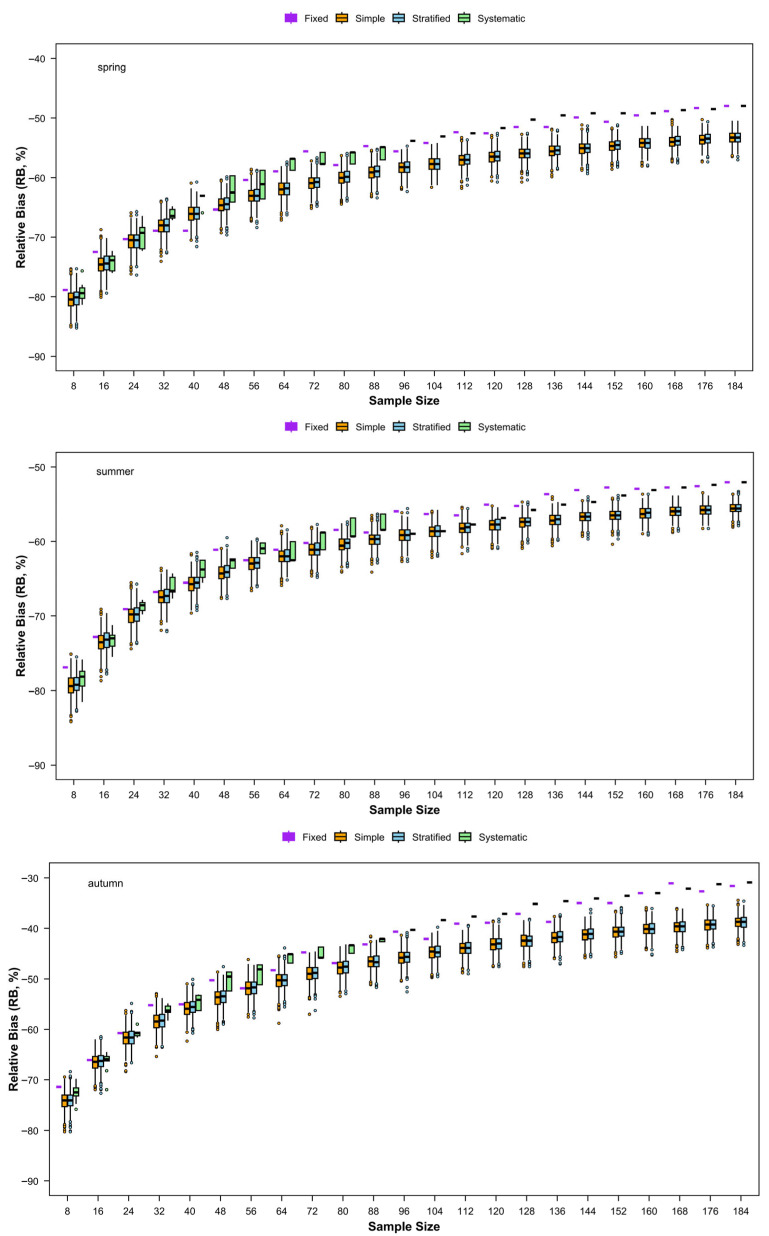

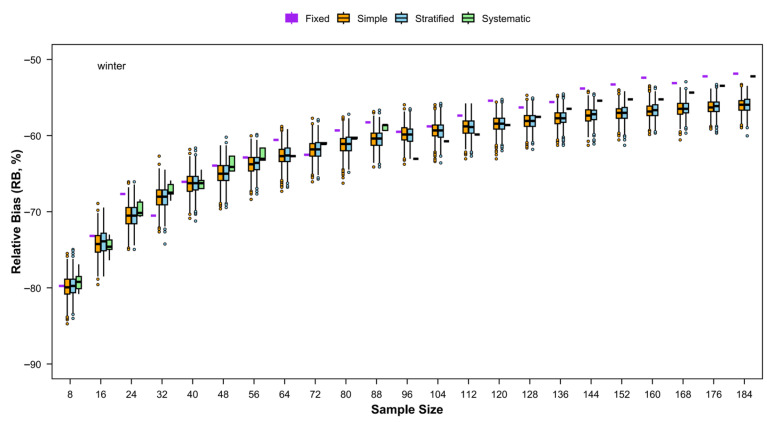
Relative bias (RB) of detection rates of fishery species with different sampling designs by sampling frequency of once per year.

**Figure A8:**
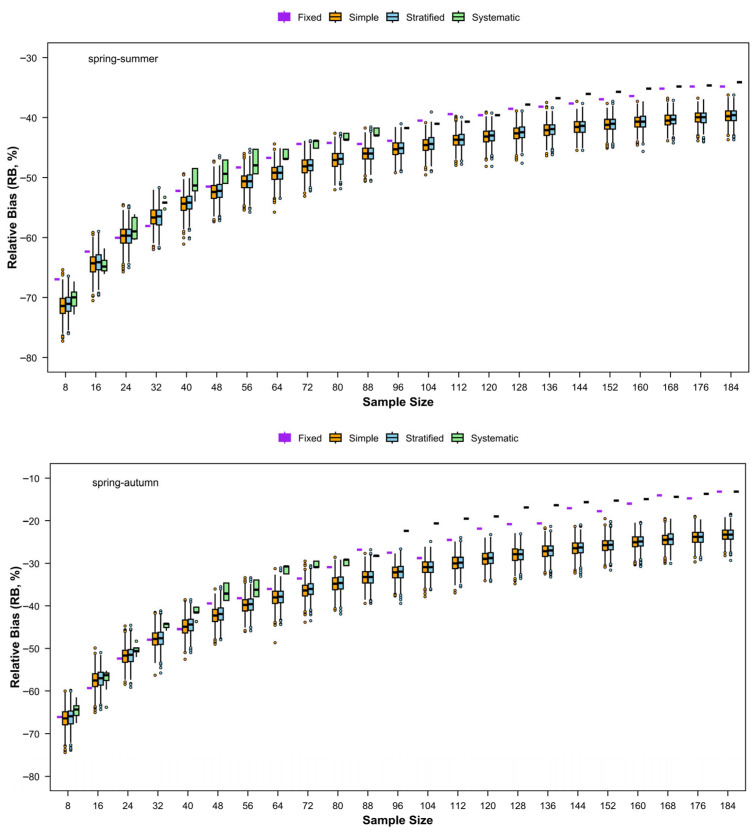

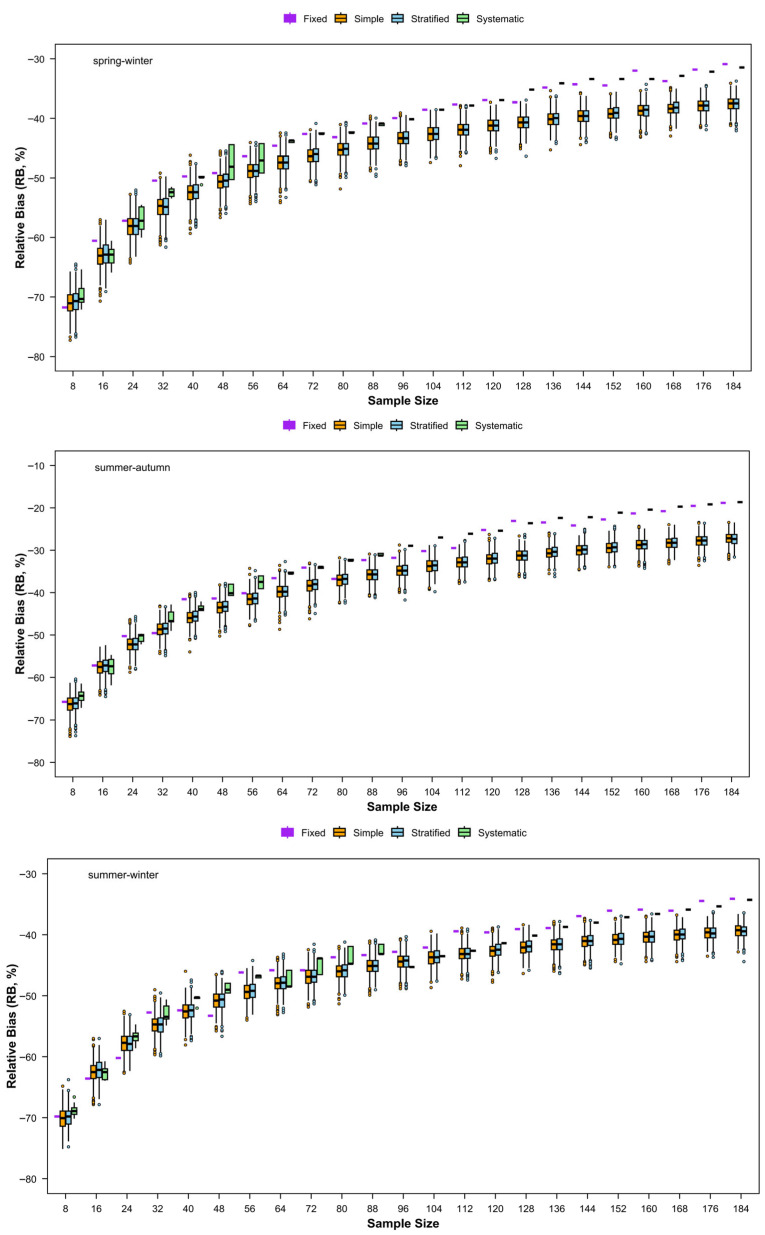

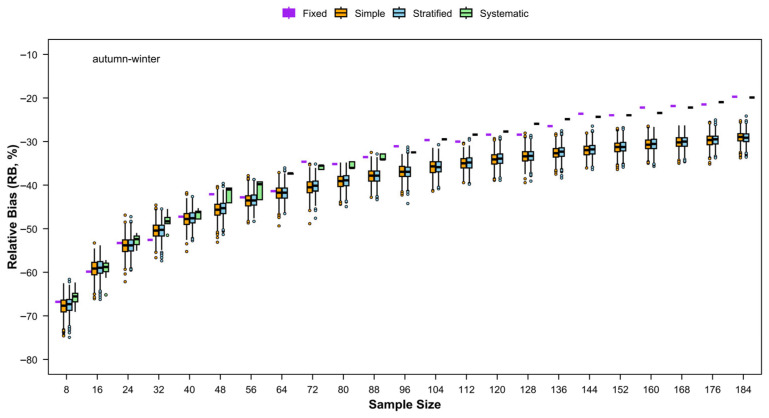
Relative bias (RB) of detection rates of fishery species with different sampling designs by sampling frequency of twice per year.

**Figure A9:**
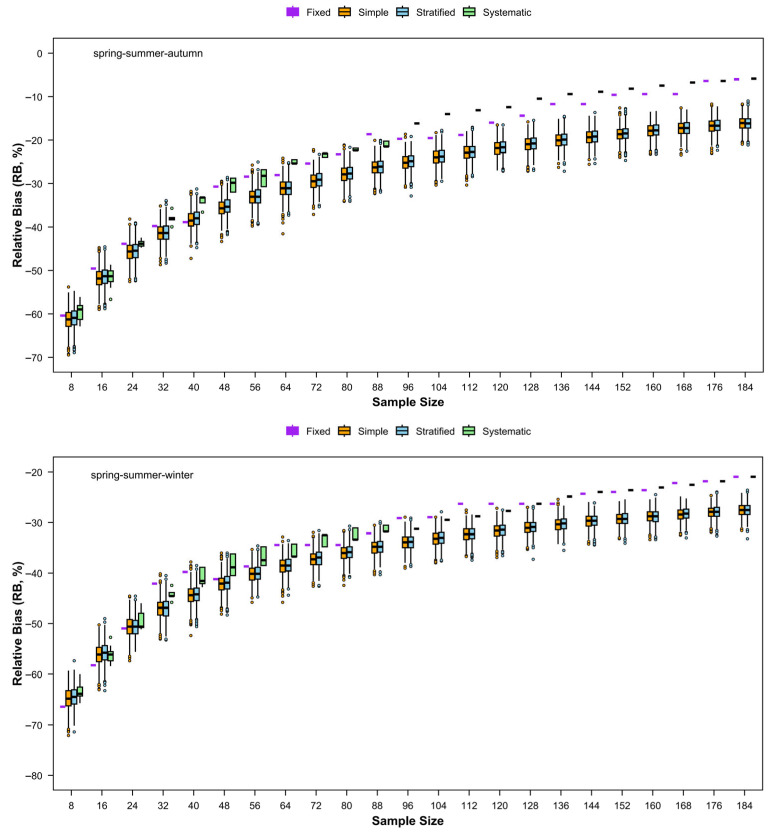

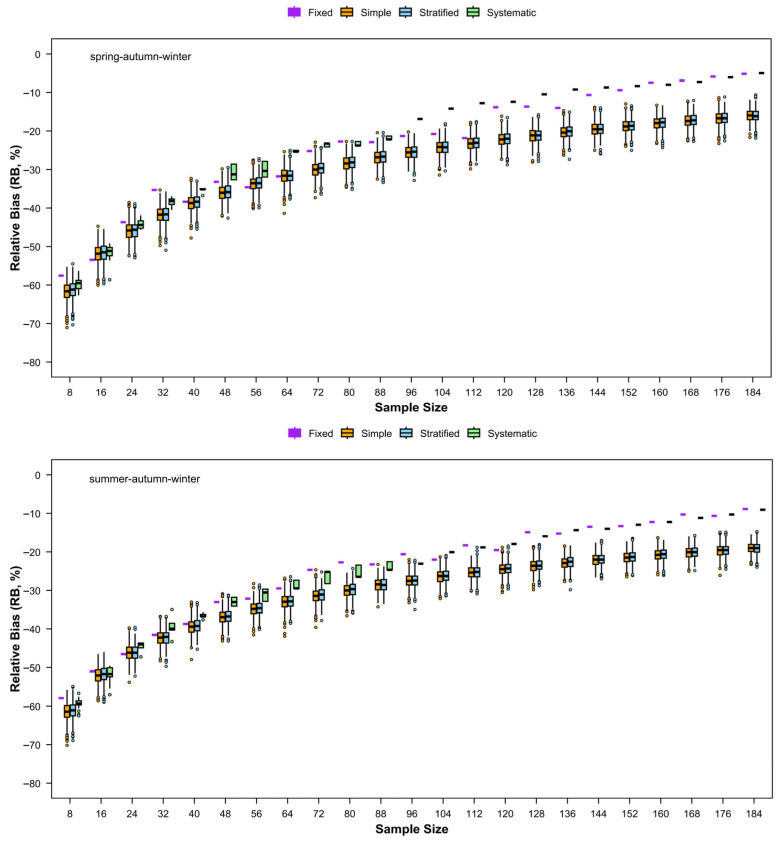
Relative bias (RB) of detection rates of fishery species with different sampling designs by sampling frequency of three times per year.

Across all sampling designs, the absolute value of relative bias (RB) for species richness detection rates decreased progressively with increasing numbers of stations (Figure 6, Appendix A Figure A7, Figure A8 and Figure A9). The decline was rapid at smaller sample sizes and then gradually leveled off. Among methods, systematic sampling yielded the smallest absolute RB, followed by fixed-site sampling, while simple random sampling had the largest. As the number of stations increased, the RB difference between stratified random sampling and simple random sampling became progressively smaller.

RB also varied with sampling frequency. For a given number of stations, higher sampling frequency led to smaller absolute RB. When sampling once, twice, and three times per year, the autumn-only (Appendix A Figure A7), spring–autumn (Appendix A Figure A8), and autumn–spring–summer (Appendix A Figure A9) combinations, respectively, produced the smallest absolute RB.

### 3.4. Relative Abundance

#### 3.4.1. REE

**Figure 7:**
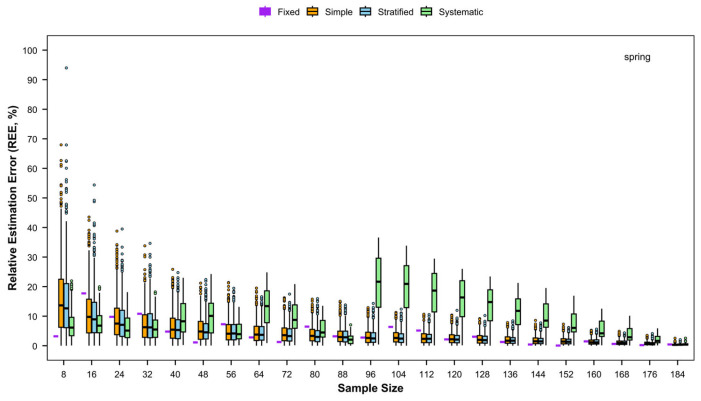

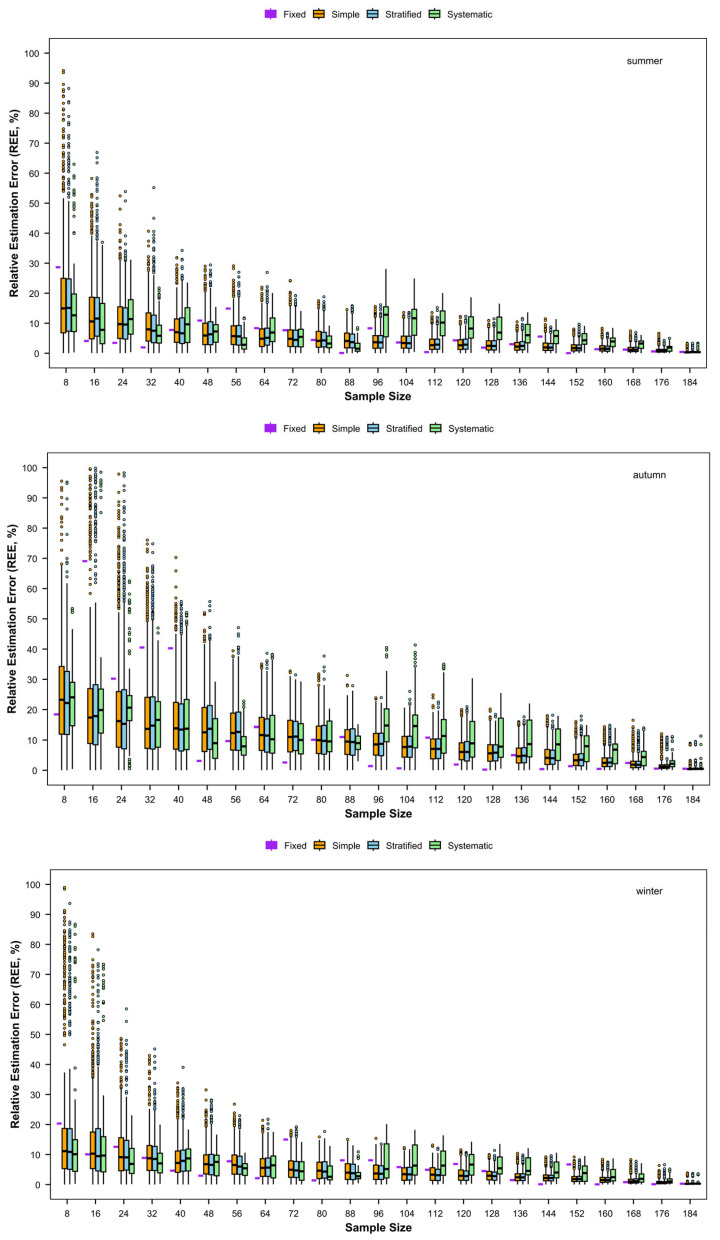
Relative estimation error (REE) of catch rates of fishery resources with different sampling designs for each season.

Distinct trends in REE for relative abundance were observed among the four sampling methods (Figure 7). For simple random sampling and stratified random sampling, REE decreased gradually with increasing numbers of stations. By contrast, REE for fixed-site sampling and systematic sampling fluctuated more markedly, generally following a pattern of initial decrease, subsequent increase, and then a further decrease. Among the methods, systematic sampling exhibited the highest REE, fixed-site samplingand simple random sampling ranked second, and stratified random sampling yielded the lowest REE.

#### 3.4.2. RB

**Figure 8:**
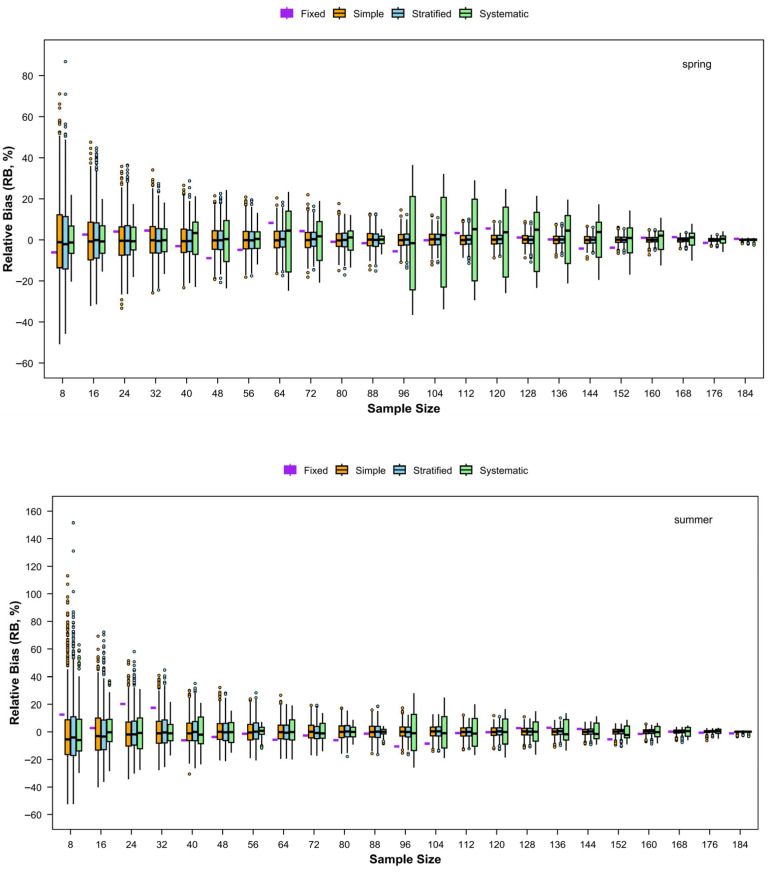

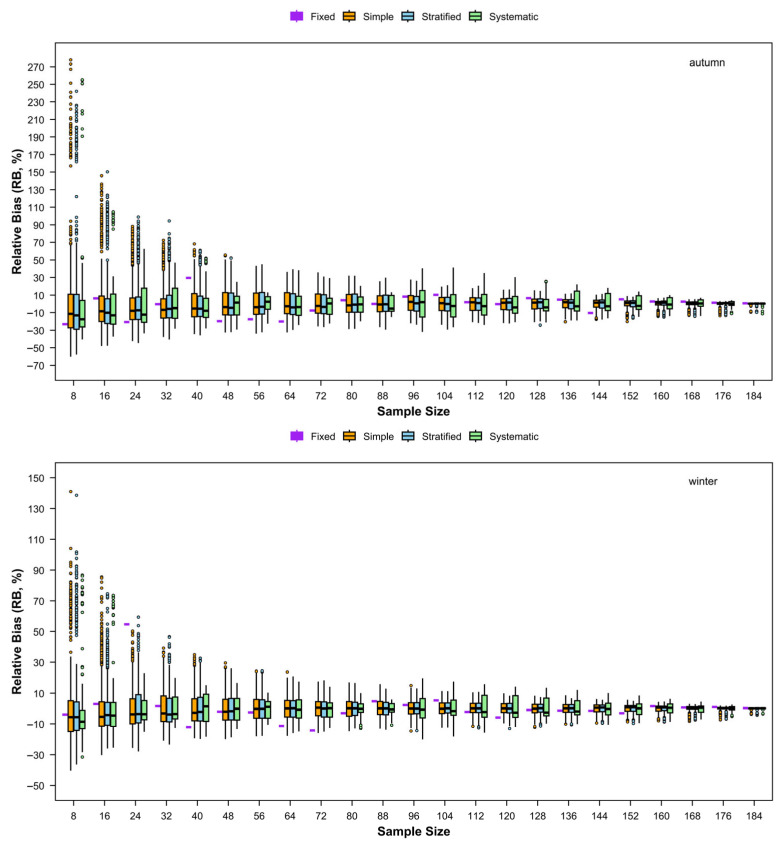
Relative bias (RB) of catch rates of fishery resources with different sampling designs for each season.

The trends in the absolute RB for relative abundance also differed among the four sampling methods (Figure 8). Across all four seasons, the absolute RB for both simple random sampling and stratified random sampling declined with increasing numbers of stations, whereas both systematic sampling and fixed-site sampling showed considerable fluctuations but an overall decreasing trend in absolute RB. In terms of absolute magnitude, systematic sampling generally exhibited the highest absolute RB, followed by stratified sampling, while fixed-site sampling resulted in the smallest absolute RB.

## 4. Discussion

This study analyzed the biodiversity and distribution of fishery resources in the restricted marine fishing area of Guangdong and provides the first systematic evaluation of how alternative sampling designs and sampling frequencies influence estimates of species richness and relative abundance. Using four seasonal bottom-trawl surveys across 186 stations, we found that: (i) species composition exhibits strong seasonality, with clear shifts in dominant taxa; (ii) catch rates vary significantly across seasons and space; (iii) species detection rates increase monotonically with station number and sampling frequency, with SS yielding the highest detection but the greatest volatility and StRS the most stable performance; and (iv) for species richness, SS minimizes REE and absolute RB compared with other methods; for relative abundance, StRS achieves the lowest REE, whereas SRS presents the smallest absolute RB.

### 4.1. The Basic Status of Fishery Resources

In this survey, the biodiversity of fishery resources (446 fish species, 101 crustacean species, and 16 cephalopod species) were comparable to those reported [31] for the entire Guangdong costal area (466 fish species, 87 crustacean species, and 21 cephalopod species). Normally, when the survey vessel and trawl gear are comparable, a larger surveyed area should yield higher fishery species richness. However, Cai et al.’s study [31] sampled only 45 stations in Guangdong’s offshore waters, whereas our study, despite covering a smaller area, set 186 stations. Thus, the two surveys reported similar numbers of fishery species.

After conversion, the mean fishery resource density in the restricted marine fishing area of Guangdong was 837.34 ± 406.83 kg/km^2^, which was higher than the density during the same period in the entire northern SCS coastal waters [32,33]. In recent years, due to multiple factors including fishing and climate change, fishery resources in the coastal waters of the northern SCS have undergone a continuous decline, with a decrease in resource density [34]. Many important commercial fish species, such as hairtail (*Trichiurus lepturus*), have exhibited size reduction and earlier sexual maturation [3]. Despite the implementation of fishery management policies such as the summer fishing moratorium, which has alleviated the pressure of overfishing and led to signs of recovery in some small-sized fish species, high trophic-level fish remain overfished [35].

Based on our results, the marine restricted fishing area has, to some extent, protected fishery resources: fishery resource density is higher than in waters outside the restricted area, and biodiversity has been maintained at a relatively high level. Nevertheless, in the waters outside the restricted zone, coordinated and targeted measures for the protection of fishery resources still need to be established to ensure their sustainable utilization.

### 4.2. Sampling Design Performance

Our results align with classical survey theory emphasizing the importance of design-based inference for accurate and robust monitoring [12,36]. Consistent with prior comparisons, we found that stratification confers notable gains in precision for abundance metrics by reducing within-stratum variance when strata are ecologically meaningful [14,15,19]. The superiority of StRS for REE in relative abundance is therefore consistent with expectations when depth-structured habitats drive assemblage composition and biomass distribution, as is typical on the northern SCS shelf [1,2], given that depth integrates multiple physiochemical gradients (e.g., light, temperature, pressure) that directly shape benthic and pelagic communities. Systematic sampling delivered the highest species detection rates and the smallest REE and absolute RB for species richness. This echoes findings that SS can outperform random designs for spatially autocorrelated targets by providing even coverage and reducing the chance of undersampling habitat patches [27,34]. However, as discussed below, this benefit can come with instability when the systematic grid coincides with environmental gradients or patch structures [14,15]. Regarding SRS, its performance was intermediate to StRS and SS for species richness, while delivering the smallest absolute RB for relative abundance. This pattern is compatible with its unbiasedness under minimal assumptions [26], but also its relatively higher variance compared to designs that exploit known structure [14]. Fixed-site sampling showed the weakest overall performance at small sample sizes—again in line with evidence that reliance on a static panel risks persistent coverage gaps and potential bias when resources and environments shift [16].

Systematic sampling enforces regular spatial spacing, which maximizes coverage and increases the probability of intersecting diverse microhabitats and rare taxa, thereby elevating species detection [37]. At the same time, when biological or environmental features are themselves periodic or strongly gradient-aligned (e.g., nearshore–offshore productivity clines, estuarine plumes, localized upwelling), a fixed interval may repeatedly sample “peaks” or “troughs,” amplifying variance across realizations and yielding performance volatility [27]. Thus, SS can be highly efficient for species discovery in patchy seascapes but sensitive to alignment with spatial structure [38].

In contrast, StRS partitions the seascape into depth-defined strata that are ecologically coherent for the region. By allocating effort proportionally across strata, StRS reduces within-stratum heterogeneity, stabilizes estimates across resamples, and yields lower REE for abundance. The observed stability advantage and precision gains are consistent with theoretical expectations and empirical syntheses [14,19]. SRS, while unbiased and easy to implement [26], forgoes the variance reduction attainable through stratification and thus tends to require larger sample sizes to match the precision of StRS. FS performed poorly at low station numbers likely because fixed panels cannot adaptively compensate for spatial shifts in hot spots and assemblage boundaries, a challenge that is exacerbated under the pronounced seasonal dynamics observed here.

### 4.3. Influence of Sampling Frequency and Seasonal Combinations

Sampling frequency exerted a strong positive effect on species detection and estimation accuracy: more frequent sampling reduced both REE and absolute RB at any given station number [39]. Among one-season designs, autumn alone performed best, and among two- and three-season combinations, spring–autumn and autumn–spring–summer, respectively, minimized error metrics. When all four seasons were sampled, only 88 stations were required to achieve an 80% detection rate.

These patterns are consistent with the seasonal ecology of the northern SCS. Autumn likely coincides with post-recruitment aggregation and transitional movements across depth bands following the summer monsoon, yielding high detectability due to broad habitat occupancy and elevated catchability for diverse taxa. The spring–autumn combination spans distinct ecological periods—spring recruitment and feeding, autumn post-monsoon redistribution—capturing complementary facets of the annual cycle and increasing the chance of encountering both early life stages and reassembled adult communities. The three-season autumn–spring–summer set further fills seasonal gaps associated with monsoon-driven hydrography (upwelling, river plume dynamics) and cross-shelf productivity shifts, explaining its efficient performance. These results corroborate the notion that survey timing relative to phenology and monsoon regimes is critical for maximizing detection and precision in monsoon-influenced shelf seas [2,3].

### 4.4. Management Implications and Recommended Survey Designs

From a cost-effectiveness perspective, our results support the following practical guidance for monitoring in Guangdong’s marine restricted fishing area. Species richness objectives: for rapid biodiversity appraisals or baselines where species discovery is prioritized, SS is attractive due to high detectability. However, to mitigate instability risks, we recommend implementing SS with random start points and periodic rotation of intervals. Additionally, incorporating safeguards—such as allocating a small portion (e.g., 10%) of the sampling effort to randomly placed stations—is crucial. This “guardrail” measure helps ensure that the sampling design does not consistently align with, and potentially miss, important environmental or biological gradients, thereby enhancing the robustness of the estimates. For routine biodiversity trend monitoring where stability and repeatability are paramount, StRS offers a balanced choice. A minimum of 88 stations across four seasons achieves an 80% detection rate; if constrained to two surveys, prioritize spring–autumn with ≥112 stations to surpass 70%.

Relative abundance objectives: StRS is preferred given its lowest REE and robustness to spatial heterogeneity in biomass. Depth-based strata used here are ecologically justified and operationally simple; proportional allocation should be maintained. If unbiasedness is prioritized under severe logistical constraints, SRS remains acceptable, noting its generally larger variance and the need for larger sample sizes to match StRS precision.

When resources allow, four-season sampling provides the best accuracy–cost trade-off (detecting 80% of species with 88 sites); otherwise, the spring–autumn pair is recommended as an alternative (requiring 112 sites to detect 70% of species). Autumn-only campaigns are suitable for minimal-effort biodiversity snapshots but should be complemented by at least one additional season when feasible to capture phenological contrasts.

### 4.5. Limitations and Future Directions

Several limitations should be acknowledged. First, only one fishing gear type (bottom trawl with a fixed cod-end mesh) was used. Gear selectivity can bias both species detection and size-specific abundance estimates, particularly for small, benthopelagic, or structure-associated taxa. Second, environmental covariates (temperature, salinity, chlorophyll, dissolved oxygen, turbidity) and dynamic drivers (river discharge, upwelling indices) were not incorporated into design optimization or post-stratification. Given the hydrographic complexity of the northern SCS [1], failing to account for these factors may limit explanatory power and obscure design–environment interactions. Third, we treated the combined species list and pooled abundance across surveys as the “true” reference, which is pragmatic but may underrepresent rare species and transient biomass pulses.

Future work should integrate environmental information into adaptive or model-assisted designs—for example, using remote sensing and ocean reanalysis products to inform dynamic stratification and allocation, and to develop spatio-temporal models that borrow strength across seasons and years [17]. Multi-gear or combined-method surveys (e.g., gill net, midwater trawl, environmental DNA, and hydroacoustics) could improve detection of pelagic, and structure-associated taxa and calibrate gear selectivity. Post-stratification by habitat classes derived from seabed maps and oceanographic fronts could further reduce variance, as it incorporates both static seabed features and dynamic water column processes, unlike the current method which relies solely on depth. It would also be valuable to test cluster or adaptive sampling schemes for rare or patchy species, and to extend the framework to adjacent shelf systems to evaluate generality. Finally, coupling survey optimization with management objectives—e.g., thresholds for recruitment indices or dominant-species monitoring—would enable explicit cost–precision trade-offs to guide long-term monitoring in Guangdong’s marine restricted fishing area [12].

Overall, by quantifying how sampling design and frequency shape biodiversity detection and abundance estimation in a spatially heterogeneous and seasonally dynamic coastal system, this study provides an evidence-based foundation for optimizing monitoring strategies that support conservation and management of fisheries resources in the northern SCS [2,36].

## 5. Conclusions

This study provides the first spatially comprehensive assessment of fishery resources and survey design performance within Guangdong’s coastal restricted fishing area. Across four seasonal bottom-trawl surveys at 186 stations, we documented high biodiversity (563 species) and pronounced seasonal and spatial variability in catch rates, with a summer peak. Species richness detection increased monotonically with station number and sampling frequency. For richness-oriented objectives, systematic sampling achieved the highest detection and the smallest REE and absolute RB but showed volatility; it should therefore be implemented with random starts and periodic interval rotation. For abundance estimation, stratified random sampling (depth-based, proportional allocation) provided the lowest REE and stable performance, while simple random sampling yielded the smallest absolute RB under tight logistical constraints. Four-season surveys required only 88 stations to reach an 80% richness detection rate; when frequency must be reduced, autumn-only (1 season), spring–autumn (2 seasons), and autumn–spring–summer (3 seasons) minimized error metrics. These results furnish practical, evidence-based guidance to balance cost and precision, supporting optimized long-term monitoring and management of fisheries in the northern SCS.

## Figures and Tables

**Table 1:** The dominant species and their IRI (the values in the brackets) in the study area for each of four seasons during 2023–2024.

Spring	Summer	Autumn	Winter
*Leiognathus brevirostris* (3372)	*Decapterus maruadsi* (6069)	*Portunus tweediei* (1751)	*Metapenaeopsis palmensis* (3928)
*Deveximentum ruconius* (3179)	*Photopectoralis bindus* (2836)	*Metapenaeopsis palmensis* (1009)	*Ilisha melastoma* (1577)
*Sardinella jussieu* (856)	*Deveximentum ruconius* (1442)	*Charybdis truncata* (916)	*Harpadon nehereus* (949)
*Saurida tumbil* (605)	*Equulites berbis* (1049)	*Ilisha melastoma* (727)	*Portunus tweediei* (716)
*Uroteuthis duvaucelii* (532)		*Deveximentum ruconius* (670)	

## Data Availability

The authors do not have permission to share data.

## Supplementary Materials

The following supporting information can be downloaded at: https://www.mdpi.com/article/10.3390/ani15223283/s1. R code for Optimizing Fishery Survey Design.

## Abbreviations

The following abbreviations are used in this manuscript:

SCS	South China Sea
FS	Fixed-site sampling
SRS	Simple random sampling
StRS	Stratified random sampling
SS	Systematic sampling
CS	Cluster sampling
REE	Relative estimation error
RB	Relative bias

## References

[1] Qiu Y., Lin Z., Wang Y. (2010). Responses of fish production to fishing and climate variability in the northern South China Sea. Prog. Oceanogr..

[2] Chen P., Qin C., Yu J., Shu L., Li X., Zhou Y., Yuan H. (2015). Evaluation of the effect of stock enhancement in the coastal waters of Guangdong, China. Fish. Manag. Ecol..

[3] Wang X., Qiu Y., Du F., Liu W., Sun D., Chen X., Yuan W., Chen Y. (2019). Roles of fishing and climate change in long-term fish species succession and population dynamics in the outer beibu gulf, south china sea. Acta Oceanol. Sin..

[4] Liu J., Gutang Q., Fan Y., Bi R., Zhao P., Zhang K., Sun Z., Li P., Liu W., Wang J. (2025). Microplastics in fish species from the eastern Guangdong: Implications to Indo-Pacific humpback dolphin (*Sousa chinensis*) and human health. Mar. Environ. Res..

[5] Wang Y., Yuan W. (2008). Changes of demersal trawl fishery resources in northern South China Sea as revealed by demersal trawling. South China Fish. Sci..

[6] MOA (2024). China Fishery Statistical Yearbooks 1978–2023.

[7] Laptikhovsky V., Allcock A.L., Barnwall L., Barrett C., Cooke G., Drerup C., Firmin C., Lozach S., MacLeod E., Oesterwind D. (2022). Spatial and temporal variability of spawning and nursery grounds of *Loligo forbesii* and *Loligo vulgaris* squids in ecoregions of Celtic seas and greater North Sea. ICES J. Mar. Sci..

[8] Johannesen E., Frøysa H.G., Langangen Ø., Vikebø F.B. (2024). Northeast Arctic haddock (*Melanogrammus aeglefinus*) spawning grounds and drift to nursery areas in the Barents Sea. Fish. Oceanogr..

[9] Cao L., Chen Y., Dong S.L., Hanson A., Huang B., Leadbitter D., Little D.C., Pikitch E.K., Qiu Y.S., de Mitcheson Y.S. (2017). Opportunity for marine fisheries reform in China. Proc. Natl. Acad. Sci. USA.

[10] Kuang T., Chen W., Huang S., Liu L., Zhou L. (2021). Environmental drivers of the functional structure of fish communities in the Pearl River Estuary. Estuar. Coast. Shelf Sci..

[11] Pan S., Lin K., Lv S., Wang X. (2022). Importance of species interactions in structuring fish community of Leizhou Bay waters, northern South China Sea. Acta Ecol. Sin..

[12] Buckland S.T., Anderson D.R., Burnham K.P., Laake J.L., Thomas L.N. (2001). Introduction to Distance Sampling: Estimating Abundance of Biological Populations.

[13] Xu B., Ren Y., Chen Y., Xue Y., Zhang C., Wan R. (2015). Optimization of stratification scheme for a fishery-independent survey with multiple objectives. Acta Oceanol. Sin..

[14] Kimura D.K., Somerton D.A. (2006). Review of statistical aspects of survey sampling for marine fisheries. Rev. Fish. Sci..

[15] Jardim E., Ribeiro P.J. (2007). Geostatistical assessment of sampling designs for Portuguese bottom trawl surveys. Fish. Res..

[16] Nelson G.A. (2014). Cluster sampling: A pervasive, yet little recognized survey design in fisheries research. Trans. Am. Fish. Soc..

[17] Delargy A.J., Cassidy K.S., Lisi A.D., Stokesbury K.D.E. (2025). A comparison of survey designs for marine benthic invertebrate sampling. Fish. Res..

[18] Folmer O., Pennington M. (2000). A statistical evaluation of the design and precision of the shrimp trawl survey off West Greenland. Fish. Res..

[19] Wang J., Xu B., Zhang C., Xue Y., Chen Y., Ren Y. (2018). Evaluation of alternative stratifications for a stratified random fishery-independent survey. Fish. Res..

[20] Zhang G., Wang J., Zhang C., Xue Y., Ren Y., Xu B. (2021). Comparison of sampling designs of fishery–independent survey in estimating abundance indices of multiple target species. J. Fish. China.

[21] Melissa T.D. (2007). Estimating sampling effort for biomonitoring of nearshore fish communities in small central Minnesota Lakes. N. Am. J. Fish. Manage..

[22] Chen D., Zhang M. (2015). Marine Fishes of China.

[23] Froese R., Pauly D. (2025). FishBase. World Wide Web Electronic Publication; Version (04/2025). https://www.fishbase.org.

[24] Pinkas L., Oliphant M.S., Iverson I.L.K. (1971). Food habits of albacore, bluefin tuna and bonito in Californian waters. Fish. Bull..

[25] Yuan M., Chen Z., Zhang J., Jiang Y., Tang Y., Xu S. (2018). Community structure of mesopelagic fish species in northern slope of South China Sea. South China Fish. Sci..

[26] Berg E. (2024). Review of the third edition of sampling: Design and analysis. J. Appl. Stat..

[27] Brown R.S. (2010). Sampling//International Encyclopedia of Education.

[28] Ma Y., Ji Y., Zhang C., Xue Y., Ren Y., Xu B. (2021). Effects of sampling design on species richness estimation of ichthyo–plankton in the coastal waters. J. Fish. Sci. China.

[29] Chen Y. (1996). A Monte Carlo study on impacts of the size of sub sample catch on estimation of fish stock parameters. Fish. Res..

[30] Paloheimo J., Chen Y. (1996). Estimating fishing mortality and cohort sizes. Can. J. Fish. Aquat. Sci..

[31] Cai Y., Xu S., Chen Z., Xu Y., Jiang Y., Yang C. (2018). Current status of community structure and diversity of fishery resources in offshore northern South China Sea. South China Fish. Sci..

[32] Cai Y., Huang Z., Xu Y., Sun M., Xu S., Zhang K., Chen Z. (2019). Probability distribution characteristics of stock density in offshore of northern South China Sea. Chin. J. Appl. Ecol..

[33] Su L., Zhang K., Xu Y., Chen Z. (2025). Variations in the fish community of the Beibu Gulf (South China Sea) following fishery resources protection measures. Fish. Res..

[34] Zhang K., Li J., Hou G., Huang Z., Shi D., Chen Z., Qiu Y. (2021). Length-based assessment of fish stocks in a data-poor, jointly exploited (China and Vietnam) fishing ground, northern South China Sea. Front. Mar. Sci..

[35] Zhang K., Su L., Chen Z., Qiu Y. (2025). An extensive assessment of exploitation indicators for multispecies fisheries in the South China Sea to inform more practical andprecise management in China. Ecol. Indic..

[36] Kotwicki S., Ono K. (2019). The effect of random and density-dependent variation in sampling efficiency on variance of abundance estimates from fishery surveys. Fish Fish..

[37] Miller T.J., Skalski J.R., Ianelli J.N. (2007). Optimizing a stratified sampling design when faced with multiple objectives. ICES J. Mar. Sci..

[38] Oyafuso Z.S., Barnett L.A.K., Kotwicki S. (2021). Incorporating spatiotemporal variability in multispecies survey design optimization addresses trade-offs in uncertainty. ICES J. Mar. Sci..

[39] Zimmermann F., Enberg K. (2016). Can less be more? Effects of reduced frequency of surveys and stock assessments. ICES J. Mar. Sci..

