# Supplementary material for: Optimizing Fishery Survey Design in Guangdong’s Restricted Coastal Waters
Source: Animals (Basel). 2025 Nov 13;15(22):3283. doi: 10.3390/ani15223283 (PMC12649134; doi:10.3390/ani15223283)
Supplement: Supplementary file 1 [file animals-15-03283-s001.zip › animals-3960571-supplementary.pdf]

R code for Optimizing Fishery Survey Design

```
library(readxl)
library(dplyr)
library(tidyr)
library(openxlsx)
library(ggplot2)
setwd("F:\\RS\\GD")
data <- read_excel("aut-win-spr-sum.xlsx")
data_long <- data %>%
  pivot_longer(-1, names_to = "Station", values_to = "Weight") %>%
  rename(Species = 1) %>%
  mutate(Weight = ifelse(is.na(Weight), 0, Weight))
layer1 <-
c("S1","S15","S25","S26","S29","S31","S68","S72","S79","S80","S83","S84","S87",
"S91","S95","S96","S101","S105","S109","S111","S112","S113","S114","S128","S1
36")
layer2 <-
c("S2","S3","S4","S5","S6","S7","S8","S9","S10","S11","S12","S14","S16","S19","S
22","S24","S28","S30","S32","S34","S35","S41","S48","S57","S61","S64","S76","S8
5","S97","S102","S110","S115","S118","S129","S137","S140","S143","S146","S150
","S153","S159","S165","S169","S170","S174","S175","S179")
layer3 <-
c("S13","S17","S20","S21","S23","S36","S37","S38","S42","S43","S49","S53","S54
","S58","S59","S65","S66","S69","S70","S73","S74","S77","S81","S88","S92","S93"
,"S98","S106","S116","S121","S125","S126","S130","S133","S134","S138","S141","
S144","S147","S148","S154","S156","S162","S163","S166","S171","S176","S180")
layer4 <-
c("S18","S27","S33","S39","S40","S44","S45","S46","S47","S50","S51","S52","S55
","S56","S60","S62","S63","S67","S71","S75","S78","S82","S86","S89","S90","S94"
,"S99","S100","S103","S104","S107","S108","S117","S119","S120","S122","S123","
S124","S127","S131","S132","S135","S139","S142","S145","S149","S151","S152","
S155","S157","S158","S160","S161","S164","S167","S168","S172","S173","S177","
S178","S181","S182","S183","S184","S185","S186")
```

```
data_long <- data_long %>%
  mutate(Layer = case_when(
    Station %in% layer1 ~ "Layer1",
    Station %in% layer2 ~ "Layer2",
    Station %in% layer3 ~ "Layer3",
    Station %in% layer4 ~ "Layer4",
    TRUE ~ NA_character_
  ))
print(table(data_long$Layer))
calculate_detection_rate_stratified <- function(data, n_samples) {
  layer_counts <- data %>%
    group_by(Layer) %>%
    summarise(n_stations = n_distinct(Station))
  samples_per_layer <- round(n_samples * layer_counts$n_stations / sum(layer_counts$n_stations))
  if(sum(samples_per_layer) != n_samples) {
    diff <- n_samples - sum(samples_per_layer)
    samples_per_layer[1] <- samples_per_layer[1] + diff
  }
  sampled_stations <- data %>%
    group_by(Layer) %>%
    distinct(Station) %>%
    sample_n(size = samples_per_layer[match(first(Layer), layer_counts$Layer)], replace = TRUE) %>%
    pull(Station)
  sampled_data <- data %>%
    filter(Station %in% sampled_stations)
  detected_species <- sampled_data %>%
    filter(Weight > 0) %>%
    pull(Species) %>%
    unique()

  detection_rate <- length(detected_species) / 563 * 100
  return(detection_rate)
```

```
}
calculate_detection_rate_simple <- function(data, n_samples) {
  sampled_stations <- data %>%
    distinct(Station) %>%
    sample_n(size = n_samples, replace = TRUE) %>%
    pull(Station)
  sampled_data <- data %>%
    filter(Station %in% sampled_stations)
  detected_species <- sampled_data %>%
    filter(Weight > 0) %>%
    pull(Species) %>%
    unique()
  detection_rate <- length(detected_species) / 563 * 100
  return(detection_rate)
}
calculate_detection_rate_systematic <- function(data, n_samples) {
  all_stations <- data %>% distinct(Station) %>% arrange(Station) %>% pull(Station)
  N <- length(all_stations)
  k <- N / n_samples
  start <- sample(1:floor(k), 1)
  sampled_indices <- sapply(1:n_samples, function(i) {
    pos <- start + (i-1)*k
    ifelse(pos > N, pos - N, pos) %>% ceiling()
  })
  sampled_stations <- all_stations[sampled_indices]
  sampled_data <- data %>% filter(Station %in% sampled_stations)
  detected_species <- sampled_data %>% filter(Weight > 0) %>% pull(Species) %>%
unique()
  return(length(detected_species) / 563 * 100)
}
sample_sizes <- c(8,16, 24, 32, 40, 48, 56, 64, 72, 80, 88, 96, 104, 112, 120, 128, 136,
144, 152, 160, 168, 176, 184)
set.seed(123)
n_simulations <- 1000
```

```
results <- data.frame(SampleSize = integer(), DetectionRate = numeric(), Method = character())
fixed_stations_list <- list()
for (n_samples in sample_sizes) {
  all_stations <- data_long %>%
    distinct(Station) %>%
    pull(Station)
  fixed_stations_list[[as.character(n_samples)]] <- sample(all_stations, size = n_samples, replace = FALSE)
  cat("size", n_samples, "set", length(fixed_stations_list[[as.character(n_samples)]]), "fixed\n")
  for (i in 1:n_simulations) {
    detection_rate <- calculate_detection_rate_stratified(data_long, n_samples)
    results <- rbind(results, data.frame(SampleSize = n_samples, DetectionRate = detection_rate, Method = "Stratified"))
  }
  for (i in 1:n_simulations) {
    detection_rate <- calculate_detection_rate_simple(data_long, n_samples)
    results <- rbind(results, data.frame(SampleSize = n_samples, DetectionRate = detection_rate, Method = "Simple"))
  }
   for (i in 1:n_simulations) {
    detection_rate <- calculate_detection_rate_systematic(data_long, n_samples)
    results <- rbind(results, data.frame(SampleSize = n_samples, DetectionRate = detection_rate, Method = "Systematic"))
  }
  fixed_stations <- fixed_stations_list[[as.character(n_samples)]]
  for (i in 1:n_simulations) {
    sampled_data <- data_long %>%
      filter(Station %in% fixed_stations)
    detected_species <- sampled_data %>%
      filter(Weight > 0) %>%
      pull(Species) %>%
      unique()
```

```
    detection_rate <- length(detected_species) / 563 * 100
    results <- rbind(results, data.frame(SampleSize = n_samples, DetectionRate =
detection_rate, Method = "Fixed"))
  }
}
write.xlsx(results, "Detection_Rates_All_Methods.xlsx", rowNames = FALSE)
p <- ggplot(results, aes(x = factor(SampleSize), y = DetectionRate)) +
  geom_boxplot(
    aes(
      color = Method,
      fill = Method
    ),
    outlier.size = 1,
    outlier.shape = 21,
    outlier.color = "black"
  ) +
  scale_color_manual(
    values = c(
      "Stratified" = "black",
      "Simple" = "black",
      "Systematic" = "black",
      "Fixed" = "purple"
    )
  ) +
  scale_fill_manual(
    values = c(
      "Stratified" = "skyblue",
      "Simple" = "orange",
      "Systematic" = "lightgreen",
      "Fixed" = "purple"
    )
  ) +
  scale_y_continuous(
    breaks = seq(0, 100, by = 10)
```

```
  ) +
  scale_x_discrete(
    breaks = levels(factor(results$SampleSize))
  ) +
  labs(
    title = "Detection Rate Comparison: Four Sampling Methods",
    x = "Sample Size",
    y = "Detection Rate (%)"
  ) +
  theme_minimal(base_size = 14) +
  theme(
panel.grid = element_blank(),
panel.border = element_rect(color = "black", fill = NA, linewidth = 0.8),
    axis.ticks = element_line(color = "black"),          axis.ticks.length = unit(0.2, "cm"),
    axis.text = element_text(color = "black"),
    axis.title = element_text(face = "bold"),
    legend.position = "top"
  )
print(p)
ggsave("Detection_Rate_All_Methods.pdf",
       plot = p,
       width = 12,
       height = 7,
       device = cairo_pdf)
```
